# Evolution of the NCI antibody-drug conjugate portfolio: from chemical engineering to clinical complexity

**DOI:** 10.3389/fmed.2026.1773414

**Published:** 2026-04-02

**Authors:** Qiang Zhan, Weilu Zeng, Haoyu Shi, Hongxia Wan

**Affiliations:** 1 Department of Breast Surgery, Pingxiang People’s Hospital, Pingxiang, China; 2 Fuzhou Medical College, Nanchang University, Fuzhou, China

**Keywords:** antibody-drug conjugates, BERTopic, immuno-oncology, National Cancer Institute, research policy, translational research

## Abstract

**Background:**

Antibody-drug conjugates (ADCs) have transcended their status as experimental pharmacophores to become a cornerstone of precision oncology. However, the trajectory of federal investment driving this clinical renaissance remains largely unmapped. Understanding the evolution of National Cancer Institute (NCI) funding priorities is critical to identifying how resource allocation has shaped—and must continue to adapt to—the shifting challenges of drug development.

**Methods:**

We performed a longitudinal analysis of the NCI grant portfolio spanning 2001 to 2025. To deconstruct the semantic evolution of the field, we employed BERTopic, a transformer-based topic modeling technique. This multi-dimensional approach correlated funding flux with institutional distribution, clinical study designs, and the technical specifications of investigated agents.

**Results:**

Our analysis delineates a distinct biphasic growth pattern, where a strategic inflection point in 2016 catalyzed an exponential surge in investment, synchronous with the clinical validation of second-generation platforms. Semantically, the field has undergone a profound structural maturation: research priorities have pivoted from foundational linker chemistry toward addressing the biological complexities of resistance mechanisms and integrating ADCs with immuno-oncology. Despite this translational momentum, we uncover a critical methodological disconnect; Analysis of experimental model systems across the full portfolio reveals that *in vivo* models represent the most prevalent explicit model type (24.0%, *n* = 94), followed by 2D cell line models (19.4%, *n* = 76), with organoid and 3D culture systems accounting for a small but growing proportion of recently funded projects (0.5%, *n* = 2). The limited adoption of organoid systems to date likely reflects the more recent emergence of these technologies rather than a deliberate funding gap.

**Conclusion:**

NCI funding has evolved in parallel with the clinical maturation of ADCs. This evolution reflects a bidirectional relationship between public investment and regulatory milestones, in which early FDA approvals catalyzed renewed federal focus on the biological bottlenecks limiting first-generation agents, while the post-2013 portfolio demonstrates a marked emergence of combination therapy and immuno-oncology integration research. However, sustaining this wave of innovation requires a strategic realignment. Future investment must bridge the identified gap in preclinical modeling fidelity, where organoid and 3D culture systems remain markedly underrepresented.

## 1 Introduction

The conceptualization of antibody-drug conjugates (ADCs) as “magic bullets”—agents capable of delivering cytotoxic payloads selectively to tumor cells while sparing healthy tissue—dates back over a century to Paul Ehrlich (1–3). However, the translation of this theoretical elegance into clinical reality has been a non-linear odyssey characterized by early setbacks, such as the immunogenicity of murine antibodies and unstable linkers, followed by recent bioengineering triumphs (4–6). Today, the field is witnessing a renaissance, catalyzed by third- and fourth-generation platforms (3, 7, 8). These advanced platforms utilize site-specific conjugation, potent topoisomerase inhibitors, and optimized drug-antibody ratios to overcome the therapeutic index limitations of their predecessors (9–13). The successful clinical implementation of recently approved agents like trastuzumab deruxtecan has redefined the standard-of-care in HER2-positive breast cancer and provided clinical validation for the “bystander effect” in overcoming intratumoral heterogeneity (14, 15). As ADCs rapidly expand into earlier lines of therapy and novel solid tumor indications, the scientific focus is shifting from primarily addressing chemical stability to tackling complex biological challenges, including mechanisms of resistance, enhancing targeted delivery, and exploring combinatorial strategies with immune checkpoint blockade (4, 11, 16–18).

The clinical trajectory of ADCs has been punctuated by a series of key FDA regulatory milestones that both reflected and shaped the direction of research investment (3, 19). Gemtuzumab ozogamicin, the first approved ADC, received FDA approval in 2000, was voluntarily withdrawn in 2010, and was reapproved in 2017 following protocol modifications (20, 21). Brentuximab vedotin was approved in 2011, followed by ado-trastuzumab emtansine in 2013. These early approvals established the clinical proof-of-concept for the ADC modality and are widely regarded as catalysts for the subsequent expansion of federal research investment in this area. A subsequent wave of approvals from 2019 onwards, including trastuzumab deruxtecan, sacituzumab govitecan, and enfortumab vedotin, has further transformed the therapeutic landscape and set the stage for the portfolio expansion documented in the present analysis (5).

Despite these clinical advances, the foundational research ecosystem driving these advances remains opaque (22). While pharmaceutical investment powers late-stage clinical trials, the National Cancer Institute (NCI) serves as the primary engine for high-risk, upstream discovery and the elucidation of fundamental mechanisms. Understanding the trajectory of federal investment is essential, as NCI funding priorities often act as a leading indicator for future therapeutic trends. Yet, the specific evolution of the ADCs portfolio remains largely uncharacterized due to the complexity of analyzing vast, unstructured grant descriptions. To truly map the “intellectual genealogy” of ADCs research, it is necessary to go beyond surface-level metrics and deconstruct the latent thematic structure of the portfolio. This allows for a precise reconstruction of how resource allocation has pivoted in response to emerging clinical data and technological bottlenecks over the past two decades.

In this study, we present a comprehensive longitudinal analysis of the NCI-funded ADCs research portfolio from 2001 to 2025. We apply BERTopic, a state-of-the-art transformer-based topic modeling technique, to extract dense semantic clusters from grant abstracts and specific aims. This approach utilizes deep learning embeddings to identify subtle shifts in research topics that manual categorization might miss (23, 24). By integrating this high-resolution semantic topology with quantitative data on funding volume, institutional distribution, and clinical study designs, we aim to examine the “black box” of translational science policy. We specifically investigate whether NCI funding has synchronized with the field’s clinical maturation, examining the transition from linker chemistry to immuno-oncology and identifying critical gaps in preclinical modeling. This multi-dimensional analysis provides a strategic roadmap for policymakers and investigators, highlighting where capital allocation has succeeded and where methodological disparities threaten to stall the next wave of therapeutic innovation.

## 2 Materials and methods

### 2.1 Data source and search strategy

Publicly available grant data were obtained from the NIH RePORTER (Research Portfolio Online Reporting Tools Expenditures and Results) database, which serves as the central repository for federally funded biomedical research. To capture the complete landscape of relevant projects, a comprehensive search strategy was executed on December 3, 2025. The dataset was strictly limited to projects funded by the NCI to isolate oncology-focused investment, with the temporal scope defined by Fiscal Years (FY) 2001 through 2025. The search query employed a Boolean logic string combining generic identifiers with specific therapeutic agents to maximize retrieval specificity. The exact search terms included: “Antibody-drug conjugate” OR “immunoconjugate”; specific approved agents and their aliases including “Trastuzumab deruxtecan” OR “T-DXd” OR “Enhertu,” “Sacituzumab” OR “Trodelvy,” “Trastuzumab emtansine” OR “T-DM1” OR “Kadcyla,” “Enfortumab” OR “Padcev,” “Brentuximab” OR “Adcetris,” “Polatuzumab,” “Mirvetuximab,” “Disitamab,” and “Gemtuzumab.” Investigational agents such as “Datopotamab” were also included. This query was applied to project titles, terms, and abstracts, yielding a raw dataset containing essential metadata including Project Serial Number, Activity Code, Project Abstract, Support Year, and Total Cost.

### 2.2 Data curation and selection criteria

Raw data were exported and processed using Python (version 3.10). To ensure the accuracy and integrity of the portfolio analysis, a multi-stage screening pipeline was implemented comprising both automated filtration and manual validation. First, to address data redundancy and prevent the artificial inflation of metrics, records were computationally deduplicated based on unique Project ID and Fiscal Year combinations. Entries representing administrative supplements or those lacking substantive abstract text were excluded. Second, to guarantee thematic precision, a manual curation phase was executed. Two independent investigators scrutinized the titles and abstracts of the retrieved records against strict inclusion and exclusion criteria. Projects were included only if they explicitly investigated the design, mechanism, preclinical evaluation, or clinical application of ADCs. Records were excluded if they focused solely on: unconjugated monoclonal antibodies; or radioimmunoconjugates (unless compared directly with ADCs). Any discordance regarding study eligibility was resolved through consensus discussion or adjudicated by a third senior reviewer. This rigorous process yielded the final corpus for downstream semantic and statistical analysis. Regarding financial metrics, grants with unlisted or redacted funding amounts were retained for project count statistics but were treated as zero values for the summation of total costs.

**FIGURE 1:**
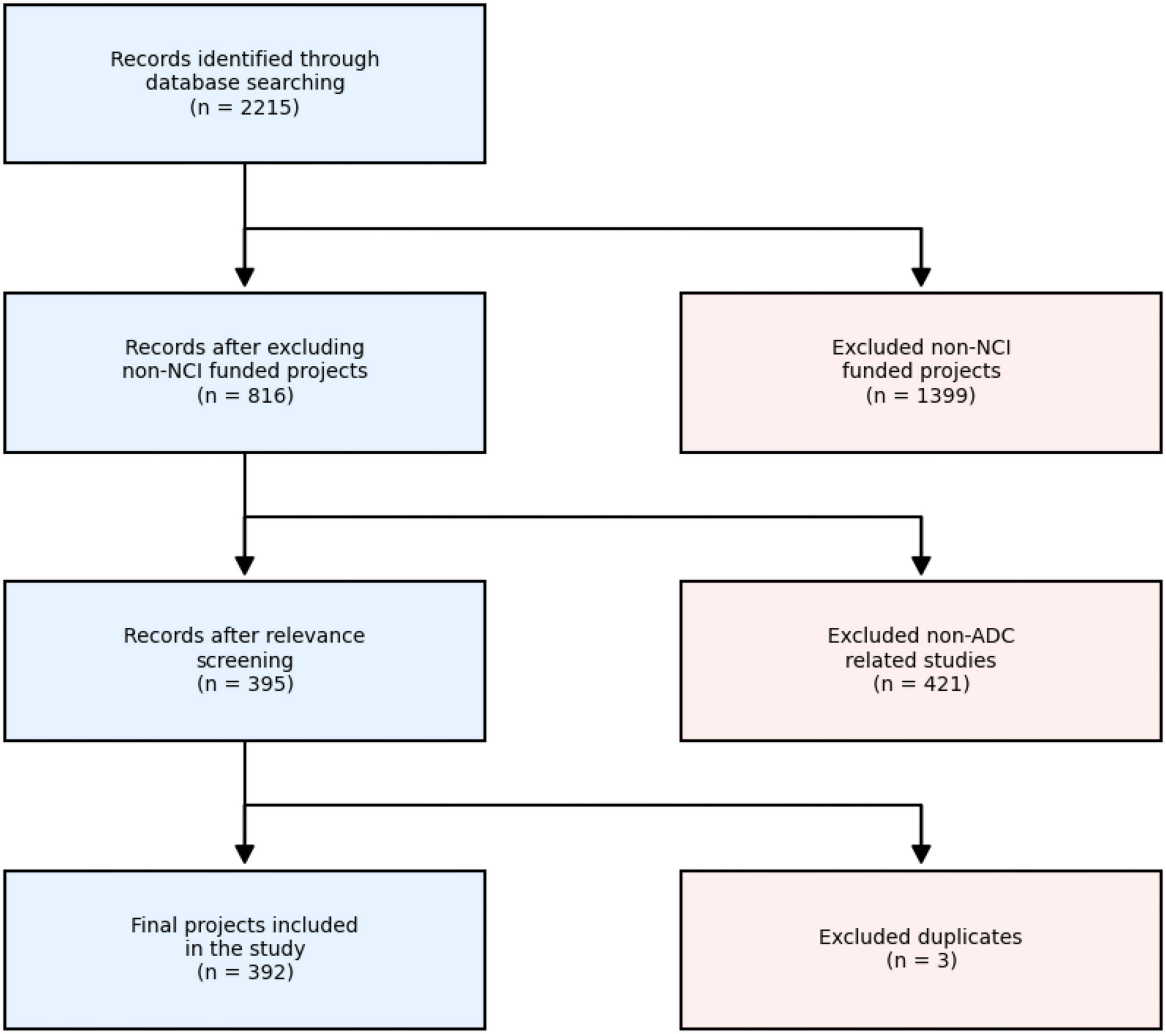
Flow diagram of the grant selection process.

A total of 2,215 records were initially identified through database searching. After excluding non-NCI funded projects (*n* = 1,399), 816 records remained. A relevance screening was then performed to remove studies not directly related to ADCs (*n* = 421) and duplicate records (*n* = 3). Finally, 392 projects were included in the portfolio analysis (Figure 1).

### 2.3 Lexicon-based semantic classification

Given that NIH grant abstracts consist of unstructured text, we developed a custom rule-based Natural Language Processing (NLP) pipeline to characterize the technical and clinical attributes of the portfolio. Project abstracts were computationally scanned using a predefined lexicon to classify grants into specific scientific domains. Payload Classification was performed by mapping abstract text against specific chemotype identifiers to distinguish between microtubule inhibitors, including maytansinoids and auristatins, and DNA-damaging agents such as topoisomerase inhibitors and pyrrolobenzodiazepine (PBD) dimers. Concurrently, Clinical Profiling involved categorizing studies by trial phase ranging from Phase I to III, and identifying specific toxicity profiles including ocular, hematological, and neurological adverse events based on keyword co-occurrence within the project descriptions. Projects were classified into four mutually exclusive study stage categories based on keyword matching applied to abstract text. Projects containing clinical trial terminology (e.g., phase I/II/III, randomized, first-in-human) without preclinical keywords were classified as “Clinical”; those containing preclinical terminology (e.g., xenograft, *in vitro*, cell line, animal model) without clinical keywords as “Preclinical”; projects containing both as “Translational/Both”; and the remainder as “Unspecified/Basic.”

### 2.4 Transformer-based topic modeling with MedCPT

To deconstruct the latent thematic structure of the NCI portfolio with high biomedical specificity, we employed BERTopic, a modular topic modeling framework. A critical deviation from standard approaches was the use of MedCPT (Medical Contrastive Pre-training) for embedding generation. Unlike general-domain language models, MedCPT is specifically pre-trained on large-scale biomedical corpora, enabling it to capture subtle semantic nuances between pharmacological mechanisms and clinical concepts that generic models might overlook (25). The modeling process involved converting grant abstracts into dense MedCPT embeddings, reducing their dimensionality using Uniform Manifold Approximation and Projection (UMAP), and clustering the data using Hierarchical Density-Based Spatial Clustering of Applications with Noise (HDBSCAN). Representative keywords for each topic were then extracted using a class-based TF-IDF procedure, allowing for the precise reconstruction of the field’s intellectual genealogy over the 25-year study period.

### 2.5 Data visualization and descriptive metrics

To characterize the longitudinal trajectory of the field, we synthesized quantitative metrics regarding funding magnitude and project volume. Total Cost was calculated as the aggregate of reported direct and indirect expenditures, utilizing nominal values to reflect actual appropriated budgets without inflation adjustment. Visual representations of these temporal trends and the distributional characteristics of funding mechanisms were generated using the Matplotlib and Seaborn libraries within the Python environment.

## 3 Results

### 3.1 Temporal evolution and thematic maturation of NCI-funded ADCs research

**FIGURE 2:**
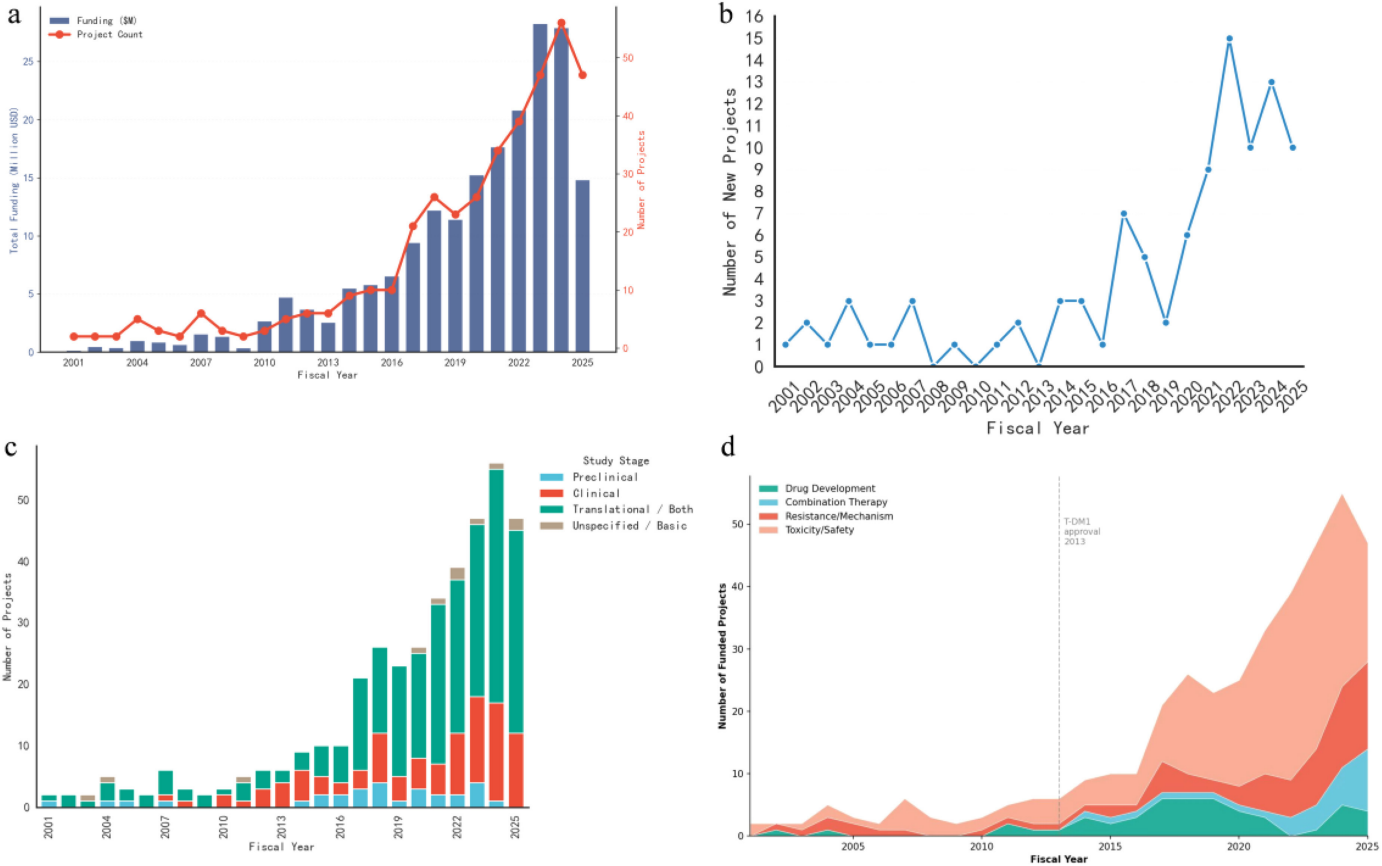
The evolving landscape of NCI-funded ADCs research (2001–2025). **(a)** Temporal trends in total annual funding (blue bars, left axis) and the annual count of funded projects (red line, right axis). Data reveal a biphasic growth pattern with a marked inflection point in 2016, leading to a synchronized surge in both funding volume and project approvals. **(b)** Annual frequency of newly initiated projects, highlighting a significant spike in new grant activations between 2019 and 2022. **(c)** Distribution of research stages over time. The portfolio shows a structural shift from unspecified/basic research (brown) in the early 2000s to a predominance of translational (green) and clinical (red) investigations in the last decade. **(d)**, Dynamic changes in research topics. Stacked area chart illustrating the thematic transition from foundational linker/chemistry studies (green) to a broader focus on mechanisms of resistance (blue), therapeutic efficacy (salmon), and toxicity/safety (pink) in recent years.

Longitudinal analysis of NCI grant data (2001–2025) delineates a distinct biphasic growth pattern in the Antibody-Drug Conjugate (ADCs) research portfolio (Figure 2). The initial phase (2001–2015) was characterized by sporadic investment and low project volume, serving as an incubation period for the field. A pivotal inflection point occurred around 2016, triggering a decade of exponential expansion where total annual funding and the number of funded projects surged synchronously, culminating in a peak allocation of over $25 million in 2023 (Figure 2a). This momentum is further corroborated by the rate of new project initiation, which exhibited a dramatic spike between 2019 and 2022, reaching a maximum of 15 new grants in a single fiscal year (Figure 2b).

Concomitant with this quantitative growth, the portfolio has undergone significant structural and thematic evolution. In terms of study stage, the landscape has transitioned from a predominance of unspecified or basic research in the early 2000s to a translational-centric model, with translational and clinical studies now comprising the majority of funded efforts (Figure 2c).

Thematic analysis of the portfolio reveals an evolving research landscape over the 25-year study period (Figure 2d). The most notable shift is the emergence of Combination Therapy research, which was virtually absent prior to 2013 but accounted for 8.8% of funded projects in 2013–2025, reflecting the growing integration of ADCs with immune checkpoint blockade strategies. Toxicity/Safety remained the dominant research focus throughout the study period, comprising 61.0% of projects in 2001–2012 and 59.8% in 2013–2025, underscoring the persistent clinical relevance of adverse event management. Resistance/Mechanism research, though representing a smaller proportional share in recent years (18.8% vs. 26.8% in the early period), has grown substantially in absolute project numbers given the overall expansion of the portfolio.

### 3.2 Institutional profile and resource allocation

**FIGURE 3:**
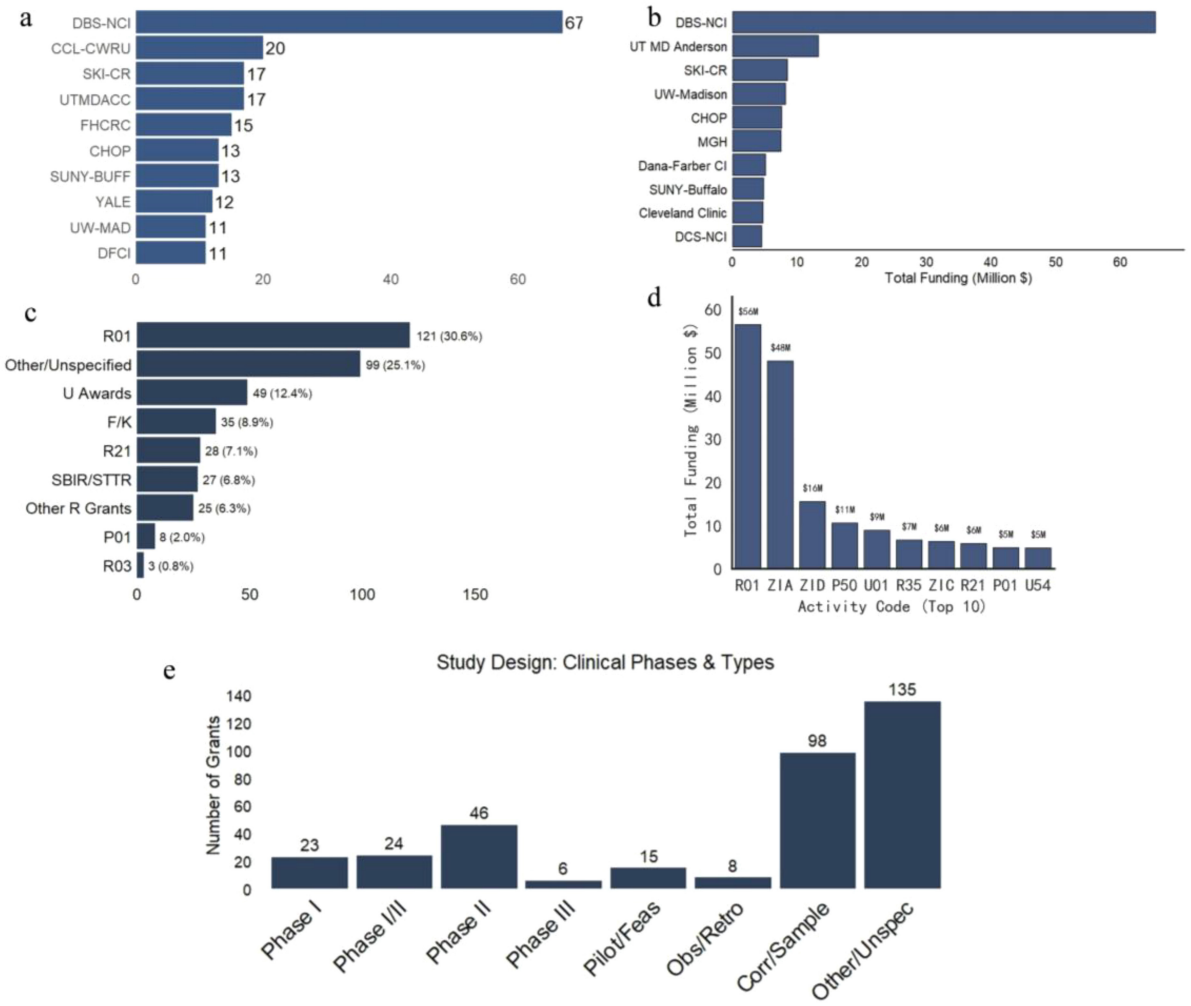
Institutional profile and structural composition of the NCI ADCs research portfolio. **(a)** Top research institutions ranked by the volume of funded projects. The NCI’s internal Division of Basic Sciences leads the field, followed by major academic cancer centers. **(b)** Leading institutions by total cumulative funding amount, illustrating the financial concentration in key research hubs. **(c)** Distribution of grant mechanisms. The Research Project Grant (R01) is the dominant funding vehicle, with substantial contributions from “Other/Unspecified” mechanisms and Cooperative Agreements (U series). **(d)** Fiscal allocation by activity code. The R01 mechanism and Intramural Research Awards (ZIA) account for the largest share of total investment. **(e)** Classification of clinical study designs. The portfolio shows a high prevalence of correlative/sample analysis studies and unspecified clinical phases, complementing traditional Phase I and Phase II trials.

We next characterized the institutional landscape and funding mechanisms underpinning the NCI’s ADCs portfolio (Figure 3). The distribution of research activity reveals a significant centralization of resources. The NCI’s internal Division of Basic Sciences ranks as the leading contributor in terms of both project volume (Figure 3a) and total cumulative funding (Figure 3b), underscoring the critical role of intramural research in this field. Among extramural institutions, major academic cancer centers such as MD Anderson Cancer Center and Sloan-Kettering Institute occupy top positions, reflecting a concentration of expertise in high-volume clinical research hubs.

Regarding funding vehicles, the Research Project Grant (R01) remains the dominant mechanism, accounting for the largest share of both grant frequency (Figure 3c) and total financial allocation (Figure 3d). This indicates a reliance on investigator-initiated, hypothesis-driven research. In terms of clinical trial design, the portfolio exhibits heterogeneity; while traditional Phase I and Phase II trials are well-represented, a substantial proportion of grants support “Correlative/Sample Analysis” or studies with unspecified phases (Figure 3e), suggesting that a significant fraction of NCI support is directed toward translational biomarker studies rather than purely interventional trials.

### 3.3 Technical specifications and clinical parameters

**FIGURE 4:**
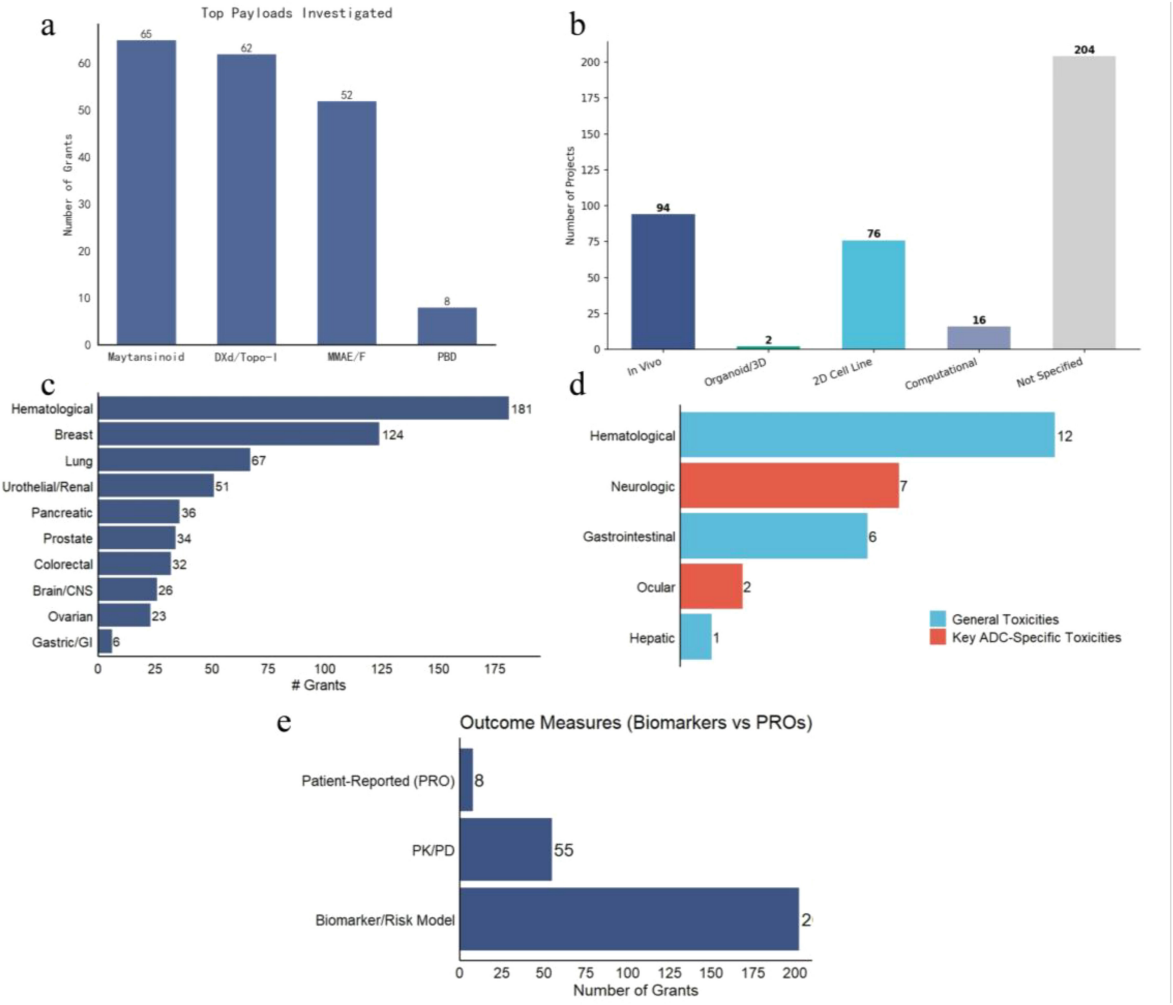
Technical specifications and clinical parameters of the NCI ADCs research portfolio. **(a)** Distribution of payload classes investigated. The field shows a balanced focus on microtubule inhibitors (Maytansinoids, MMAE/F) and topoisomerase inhibitors (DXd/Topo-I), PBD: pyrrolobenzodiazepine. **(b)** Distribution of experimental model systems across the full portfolio (*n* = 392, mutually exclusive classification). Projects are assigned to the highest-fidelity model category mentioned in the abstract: *In Vivo*, Organoid/3D, 2D Cell Line, Computational, or Not Specified. **(c)** Disease indications ranked by grant volume. Hematological malignancies constitute the primary area of research, with Breast and Lung cancer leading among solid tumors. **(d)** Classification of specific toxicities studied. Hematological and neurological toxicities are the primary safety concerns addressed in funded projects. **(e)**, Frequency of outcome measures. The portfolio is heavily skewed toward Biomarker/Risk Model development, with limited representation of Patient-Reported Outcomes (PROs).

We further characterized the technical composition and clinical focus of the NCI-funded ADCs portfolio. In terms of payload chemistry, the research landscape is dominated by three major classes: Maytansinoids, Topoisomerase I inhibitors (DXd/Topo-I), and Auristatins (MMAE/F), with Maytansinoids and DXd showing comparable prevalence (Figure 4a). Analysis of experimental model systems across the full portfolio (*n* = 392) reveals that the largest proportion of projects did not specify a particular model system in their abstract (52.0%, *n* = 204), reflecting the substantial representation of clinical and translational studies in the portfolio (Figure 4b). Among projects employing explicit experimental models, *in vivo* systems were most prevalent (24.0%, *n* = 94), followed by 2D cell line models (19.4%, *n* = 76), computational approaches (4.1%, *n* = 16), and organoid/3D systems (0.5%, *n* = 2). The two organoid/3D projects were both funded after 2018, consistent with the broader adoption of these technologies in oncology research in recent years.

Clinically, the portfolio reflects the current landscape of ADCs approval and development. Hematological malignancies represent the largest disease focus, followed by breast and lung cancers among solid tumors (Figure 4c). Safety profiling indicates that hematological toxicity is the most investigated adverse event, followed by neurological and gastrointestinal toxicities (Figure 4d). Furthermore, when evaluating study endpoints, there is a predominant emphasis on biomarker discovery and risk modeling (Figure 4e), which significantly outnumbers studies focused purely on pharmacokinetics/pharmacodynamics (PK/PD) or patient-reported outcomes (PROs).

### 3.4 Semantic topology and thematic evolution

**FIGURE 5:**
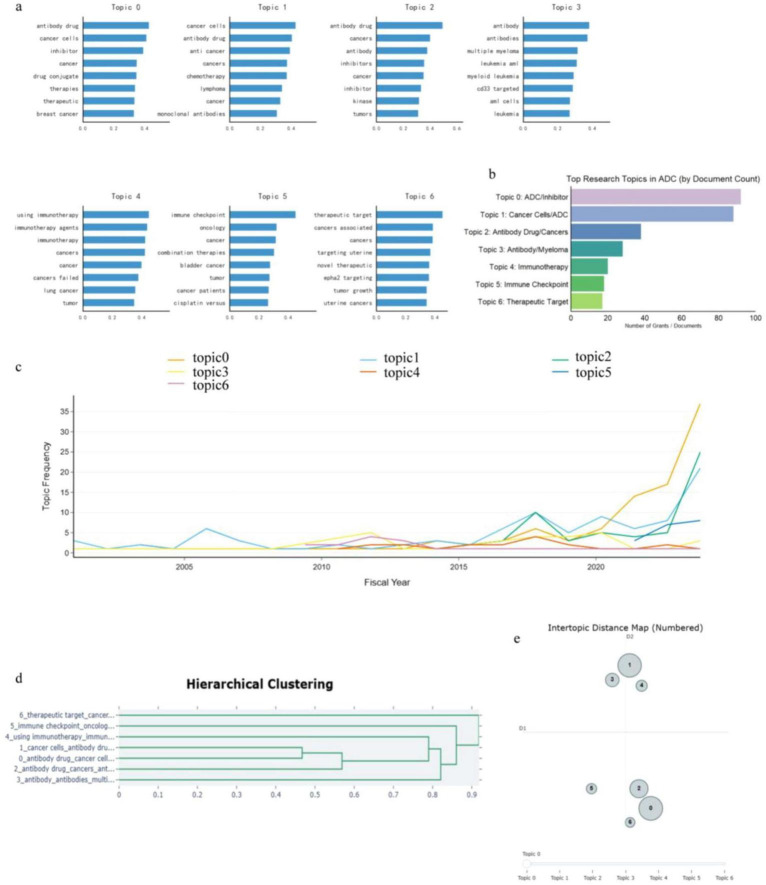
Latent topic modeling and semantic evolution of the NCI ADCs grant corpus. **(a)** Top representative keywords associated with each of the seven identified latent topics. **(b)** Distribution of research topics by document count. The portfolio is numerically dominated by general mechanistic themes (Topics 0–2), with specific clusters emerging for multiple myeloma (Topic 3) and immunotherapy (Topic 4). **(c)** Temporal dynamics of topic frequency. The trajectories highlight a recent surge in research output related to therapeutic targets (yellow line) and immune-oncology intersections relative to stable historical baselines. **(d)** Hierarchical clustering dendrogram illustrating semantic relationships. The structure reveals a dichotomy between foundational mechanism-focused topics (Topics 0–2) and the distinct cluster of immunotherapy/target-focused topics (Topics 4–6). **(e)** Intertopic distance map visualized via multidimensional scaling (MDS). The spatial separation confirms the semantic independence of the identified research clusters within the grant portfolio.

To deconstruct the underlying thematic structure and semantic evolution of the NCI-funded portfolio, we employed BERTopic, a transformer-based topic modeling technique that uses deep learning embeddings (Figure 5). This analysis identified seven distinct latent topics characterizing the corpus (Figure 5a). In terms of prevalence, the field is anchored by foundational themes (Topics 0 and 1) related to general antibody-drug mechanisms and cancer cell inhibition, which account for the largest volume of documents (Figure 5b).

The BERTopic analysis identifies seven semantically distinct research clusters within the NCI ADC portfolio. While Topics 0–2 are anchored by foundational ADC mechanistic vocabulary shared across the corpus, Topics 4 and 5 are specifically characterized by immunotherapy-related terminology—including immunotherapy agents, immune checkpoint, and combination therapies—that is largely absent from the foundational topics. This semantic distinction provides quantitative evidence for the emergence of a discrete immuno-oncology–oriented research cluster within the portfolio, reflecting the growing integration of ADCs with immune checkpoint blockade strategies. Topic 3 captures a focused hematological malignancy cluster centered on AML and multiple myeloma, while Topic 6 reflects the emergence of novel therapeutic targeting approaches including EphA2. The full keyword profiles for each topic are as follows: Topic 0 (General ADC Mechanisms: antibody drug, cancer cells, inhibitor, drug conjugate, therapies), Topic 1 (Cytotoxic Combination: cancer cells, anti-cancer, chemotherapy, lymphoma, monoclonal antibodies), Topic 2 (Targeted Inhibition: antibody drug, inhibitors, kinase, tumors), Topic 3 (Hematological Malignancies: antibody, multiple myeloma, leukemia AML, CD33), Topic 4 (Immunotherapy Integration: immunotherapy, immunotherapy agents, cancers failed, lung cancer), Topic 5 (Immune Checkpoint Combination: immune checkpoint, oncology, combination therapies, bladder cancer), and Topic 6 (Novel Target Discovery: therapeutic target, novel therapeutic, EphA2 targeting, uterine cancers).

Temporal analysis (Figure 5c) reveals the evolutionary trajectory of these themes: while foundational topics remain consistent, there is a pronounced acceleration in topics associated with novel therapeutic targets and immunomodulation from 2015 onwards.

The hierarchical clustering dendrogram (Figure 5d) suggests two higher-level semantic groupings. Topics 4 and 5 cluster most closely (immunotherapy integration and immune checkpoint–related combinations), with Topic 6 (therapeutic/target discovery) joining this immuno-oncology–adjacent cluster before it merges with Topics 0–2, which group together more tightly and reflect broader foundational mechanistic ADC themes. Topic 3 (hematological malignancies) forms a comparatively independent branch that joins the other topics at a higher linkage distance. This structural separation is further reflected in the intertopic distance map (Figure 5e), in which Topics 1, 3, and 4 co-occupy the positive D2 quadrant, while Topics 0, 2, and 6 cluster in the negative D2 quadrant. Topic 5 occupies a comparatively isolated position in the lower-left region, suggesting greater semantic distinctiveness from the remaining topics. Taken together, these two complementary visualizations are consistent with a semantic separation between general cytotoxic/mechanistic themes and more immune-interactive or target-oriented ADC research, while we emphasize that both hierarchical clustering and multidimensional scaling reflect similarity structure rather than temporal or causal relationships.

## 4 Discussion

This longitudinal semantic analysis of the NCI grant portfolio reconstructs the twenty-five-year intellectual genealogy of ADCs, revealing a distinct synchronization between federal investment and the modality’s clinical renaissance. By leveraging domain-specific contrastive learning (MedCPT) to deconstruct the latent structure of grant texts, we mapped a fundamental evolutionary trajectory: the field has decisively pivoted from an early preoccupation with linker stability and microtubule-targeting payloads to a contemporary focus on DNA-damaging agents (specifically topoisomerase inhibitors), mechanisms of resistance, and combinatorial strategies with immune checkpoint blockade. Our data are consistent with a pattern in which NCI funding concentrated on upstream mechanistic research themes, including targeted inhibition and immunotherapy integration, during a period that preceded the broader clinical validation of these approaches. However, we acknowledge that our analysis is limited to NCI grant abstracts and does not include pharmaceutical R&D investment data; accordingly, a direct causal comparison between public and private sector funding priorities cannot be established from the present dataset. These findings highlight the critical role of upstream public funding in validating the molecular principles that underpin the recent wave of practice-changing regulatory approvals.

While prior bibliometric analyses have successfully charted the exponential growth of ADCs publications and patent landscapes (22, 26, 27), these studies rely predominantly on publication outputs—lagging indicators that reflect research initiated years prior. Our findings align with the consensus in these retrospective studies regarding the broad industrial pivot from hematological malignancies to solid tumors (28). However, by analyzing upstream federal funding through the lens of MedCPT embeddings, our study reveals a critical divergence from traditional keyword-based metrics. Where conventional analyses suggest a linear technological progression, our data uncover a more complex, NCI-driven “de-risking” phase. The seven identified topics reflect distinct but complementary research themes within the NCI ADC portfolio. Topics 0–2 capture foundational mechanistic research encompassing general ADC mechanisms, cytotoxic combinations, and targeted inhibition. Topic 3 delineates a focused cluster on hematological malignancies, particularly AML and multiple myeloma. Topics 4 and 5 reflect the growing integration of ADCs with immunotherapy and immune checkpoint blockade, while Topic 6 highlights the emergence of novel therapeutic targeting strategies including EphA2. This indicates that NCI funding did not merely react to the success of third-generation agents like trastuzumab deruxtecan but actively established their preclinical foundation by prioritizing high-risk mechanistic research—such as optimizing the bystander effect—long before these concepts became standard commercial viability markers, thereby paving the way for the ongoing development of fourth-generation platforms (29).

Crucially, our semantic topology highlights a fundamental reconceptualization of the ADCs mechanism of action—from targeted chemotherapy delivery to an immunomodulatory “payload-first” approach (30). The distinct funding migration from microtubule-disrupting agents to DNA-damaging payloads, particularly topoisomerase I inhibitors, reflects a strategic pivot to address the limitations of antimitotics in heterogeneous, slow-cycling solid tumors (31). This NCI prioritization anticipated the clinical field validated by trastuzumab deruxtecan, where the payload’s membrane permeability and subsequent “bystander killing” proved essential for overcoming target heterogeneity (31). Furthermore, the rapid expansion of the “Immuno-oncology Integration” cluster indicates that federal investment is increasingly treating ADCs as inducers of immunogenic cell death rather than monotherapies, aiming to sensitize “cold” tumor microenvironments to checkpoint blockade (32). This trajectory suggests that the next generation of ADCs will be defined not merely by linker stability, but by their capacity to synergize with host immunity and overcome resistance mechanisms driven by antigen loss (18, 33).

Finally, we acknowledge certain limitations to this study. Primarily, our analysis is restricted to NCI-funded projects, thereby excluding private pharmaceutical R&D expenditures and international investments, which are substantial drivers of the global ADCs landscape. Additionally, grant funding represents a leading indicator of research intent rather than immediate output, implying a temporal lag between capital allocation and disseminated clinical results. Technologically, while MedCPT embeddings offer superior semantic resolution compared to keyword matching, algorithmic topic modeling remains a probabilistic approximation that may occasionally miss subtle contextual nuances.

Another important limitation of the present study is that the upstream funding data analyzed here are not integrated with downstream metrics such as patent filing activity, FDA regulatory approval timelines, or real-world clinical outcomes from registry databases. Accordingly, the findings should be interpreted as reflecting shifts in public funding priorities rather than providing a complete assessment of translational efficiency or real-world impact. Readers should bear in mind that the observed trends in NCI project funding do not, in themselves, speak to whether those investments have yielded—or are likely to yield—commensurate advances in clinical practice, and a comprehensive evaluation of public investment strategies in precision oncology would require the incorporation of such downstream indicators.

## 5 Conclusion

The longitudinal semantic reconstruction of the NCI grant portfolio serves as a definitive historical record of the maturation of ADCs from theoretical “magic bullets” to clinically validated biological platforms. Our analysis demonstrates that federal resource allocation has acted as a strategic radar, anticipating the necessity of high-potency DNA-damaging payloads and the exploitation of the bystander effect long before these mechanisms revolutionized the standard of care. The distinct trajectory—from defining chemical linker stability to unraveling complex mechanisms of resistance—suggests that the next generation of ADCs will be defined not by their structural composition alone, but by their integration into the broader immuno-oncology ecosystem. As the demarcation between cytotoxic chemotherapy and immunotherapy continues to blur, future policy frameworks must prioritize interdisciplinary research that bridges chemical biology with tumor immunology. Methodologically, the transition from keyword-based indexing to deep semantic modeling offers a replicable framework for mapping translational science. Future inquiries should aim to synthesize upstream funding data with downstream global patent landscapes and real-world clinical outcomes. Ultimately, establishing such an end-to-end evidence model will be essential for optimizing public investment strategies and accelerating the delivery of precision therapeutics to patients.

## 6 Limitations of the study

Several limitations of this study warrant acknowledgment. First, our analysis is restricted to NCI-funded projects and does not capture private pharmaceutical R&D or international investments, limiting the generalizability of the observed trends to federal public funding priorities. Second, research classification was performed using automated keyword matching on publicly available abstracts rather than manual review of complete grant documents, which may result in conservative estimates. Third, the priority-based classification schemes prevent double-counting but may underestimate themes that co-occur within a single abstract. Fourth, grant funding represents a leading indicator of research intent rather than immediate output, and a temporal lag exists between capital allocation and clinical results. Future investigations should integrate upstream funding data with downstream metrics including patent filings, regulatory timelines, and real-world clinical outcomes.

## Data Availability

The original contributions presented in the study are included in the article/supplementary material, further inquiries can be directed to the corresponding author.
